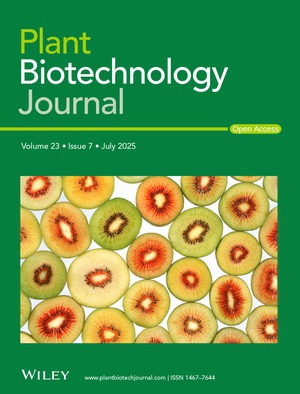# Issue Information

**DOI:** 10.1111/pbi.14393

**Published:** 2025-06-28

## Abstract

Front cover image:

Color change in kiwifruit occurs during fruit ripening and is usually triggered by the degradation of green chlorophyll concomitant with the accumulation of pigments, such as red anthocyanins and yellow carotenoids. The study reveals that degreening in kiwifruit occurs via a regulatory cascade involving the NAC transcription factors *AcNAC2* and *AcNAC3* and the Mg‐dechelatases *AcSGR1* and *AcSGR2*.